# Bispecific Siglec-15/T cell antibody (STAB) activates T cells and suppresses pancreatic ductal adenocarcinoma and non-small cell lung tumors *in vivo*

**DOI:** 10.7150/thno.103372

**Published:** 2025-04-13

**Authors:** Limei Shen, Alison Schaefer, Justin Huckaby, Whitney Wolf, Samuel K. Lai

**Affiliations:** 1 Division of Pharmacoengineering and Molecular Pharmaceutics, Eshelman School of Pharmacy, University of North Carolina at Chapel Hill, NC, USA.; 2 Department of Biomedical Engineering, University of North Carolina at Chapel Hill, NC, USA.; 3 Lineberger Comprehensive Cancer Center, University of North Carolina at Chapel Hill, NC, USA.; 4 Department of Immunology and Microbiology, University of North Carolina at Chapel Hill, NC, USA.

**Keywords:** bispecific T cell engager (biTE), tumor microenvironment, immunotherapy

## Abstract

**Rationale:** Siglec-15 (S15) is a membrane-associated antigen overexpressed across various cancer types, and also induces immunosuppression. We believe this makes S15 a promising target for cellular immunotherapy of solid tumors characterized by an immunosuppressive tumor microenvironment, but this remains underexplored to date.

**Method:** We engineered a bispecific antibody that simultaneously binds S15 on tumor cells and CD3 on T cells in the popular IgG-scFv format; we termed this molecule STAB.

**Results:** *In vitro*, STAB induced marked proliferation of CD3+ T cells in human PBMCs, and mediated effective killing of Panc-1 pancreatic ductal adenocarcinoma (PDAC) and H460 non-small cell lung cancer (NSCLC) cells in co-culture studies with PBMCs or CD3+ T cells. In NSG mice with human PDAC and NSCLC tumors, STAB effectively suppressed tumor growth and prolonged survival, in sharp contrast to mice receiving either anti-S15 or anti-CD3 mAbs alone. STAB increased activated T cells in both tumor and circulation, as well as reduced the stromal barrier—a key hallmark of PDAC.

**Conclusion:** Our results underscore STAb as a promising therapeutic molecule to be investigated further for PDAC and NSCLC, and potentially other S15-positive solid tumors.

## Introduction

Pancreatic ductal adenocarcinoma (PDAC) is a highly aggressive cancer with a dismal five-year overall survival rate of just 6% 1, and few promising interventions on the horizon. Despite a far lower incidence rate than other malignancies, PDAC was the third highest cause of cancer-related death in 2021, and is projected to become the second most frequent cause of cancer-related death by 2030 2. PDACs' poor prognosis is attributed to the difficulty in early diagnosis at a curable stage: patients rarely exhibit symptoms early on, and PDACs typically do not display sensitive and specific markers that aid early detection 3. PDACs also have few prevalent genetic mutations, and none of the most commonly mutated genes in PDAC - KRAS, CDKN2A (encoding p16), TP53 and SMAD4 — are currently 'undruggable' 4. By the time a diagnosis is made, the dense and rich stroma surrounding the tumor and the immunosuppressive tumor microenvironment (TME) strongly restricts the distribution of various therapeutics into the tumor as well as limits tumor-specific immunity 5. As a result, PDAC is refractory to most classes of therapies 6, 7, and clinical efficacy with most checkpoint inhibitors and immune cell therapies such as CAR-T remains modest at best 8.

Similar to PDAC, lung cancer, in particular non-small cell lung cancer (NSCLC), is the leading cause of cancer mortality worldwide accounting for ~25% of all cancer-related mortality 9. The poor prognosis is again attributed to the lack of biomarker available for early detection that results in diagnosis of majority of NSCLC only at an advanced state. By the time treatment can be initiated, NSCLC already possess a highly immunosuppressive TME 10 as well as a dense stromal barrier 11, both contributing to making NSCLC tumors highly refractory to available treatments, including checkpoint inhibitors 12 and CAR-T therapies 13. Indeed, unselected advanced NSCLC patients typically respond to existing treatments only ~20% of the time 14.

To improve immunotherapy for solid tumors such as PDAC and NSCLC, we believe we must simultaneously target an antigen broadly overexpressed in the relevant cancer tissues, while also addressing the immunosuppressive TME. This led us to Siglec-15 (S15), a member of the Siglec family expressed at high levels in a wide variety of tumor cells and tumor-associated macrophages, at low levels in circulating monocytes, and otherwise minimally expressed in various healthy tissues 15, 16. Interestingly, S15 has similar immunosuppressive functions as PD-L1, and its expression appears to be mutually exclusive of PD-L1, via a mechanism-of-action that appears to be distinct from the PD-1/PD-L1 axis 16. This suggests targeting S15 may be particularly useful against tumors that are non-responsive to anti-PD-1/PD-L1 therapy. We hypothesize that molecules that bind both S15 and CD3 on T cells may serve the dual purpose of better homing T-cells to S15+ tumors, while also reducing the immunosuppression within the TME that limits the efficacy of T-cell immunity against most solid tumors. Here, we report on the engineering of a bispecific S15/CD3 antibody (STAB) and its promising efficacy against both PDAC and NSCLC *in vivo*.

## Results

### S15 expression in both PDAC and NSCLC tissues

**Figure 1:**
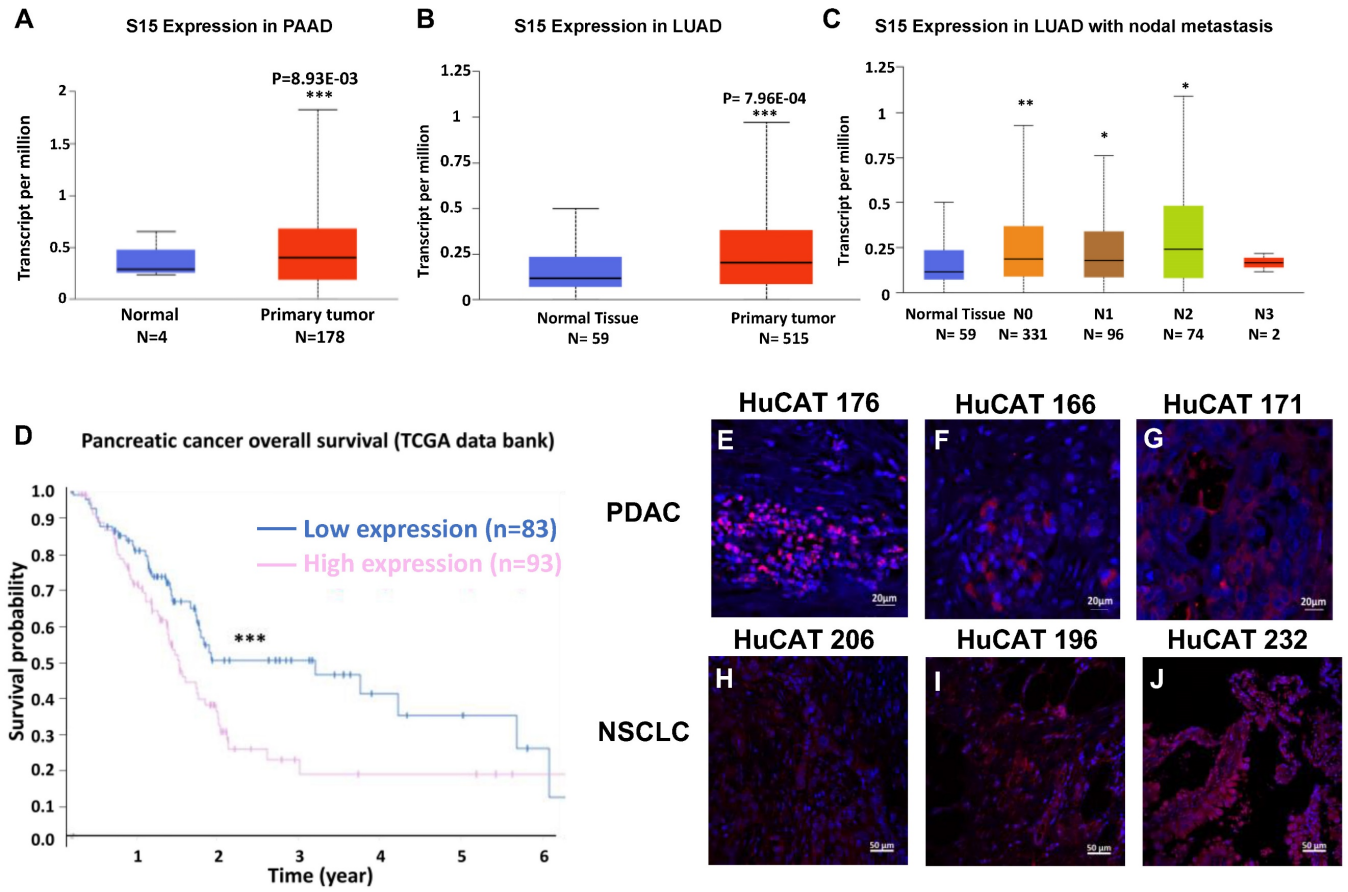
** Effect of S15 overexpression on survival in PDAC and NSCLC patients and detection of S15 in tumor tissues**. **(A)** S15 is overexpressed in PDAC patients, as shown by data from TCGA. **(B-C)** S15 is also overexpressed in NSCLC patients, including those with metastatic disease (data from TCGA). **(D)** Kaplan-Meier survival analysis indicates that pancreatic cancer patients with high S15 expression exhibit significantly lower overall survival (data from TCGA). **(E-G)** S15 expression was detected in pancreatic cancer patient tumor tissues by immunohistochemistry (IHC) staining. Primary anti-human S15 antibody was used at a 1:500 dilution (0.9 mg/mL), and secondary rabbit anti-human Alexa594 antibody was used at a 1:1000 dilution for detection. **(H-J)** S15 expression was also detected in NSCLC patient tumor tissues using the same staining protocol with anti-human S15 and rabbit anti-human Alexa594 antibodies.

We first sought to confirm the overexpression of Siglec-15 (S15) in both pancreatic ductal adenocarcinoma (PDAC) and non-small cell lung cancer (NSCLC). Data from The Cancer Genome Atlas (TCGA) program show significant overexpression of S15 mRNA in pancreatic cancer (**Figure 1A**), lung cancer (**Figure 1B**), and metastatic lung cancer samples (**Figure 1C**). This overexpression aligns with our previous findings in triple-negative breast cancer, as well as with studies conducted by Lieping Chen's group 16. Elevated S15 expression is associated with a generally poor prognosis in both PDAC (**Figure 1D**) and NSCLC 17. We validated S15 expression in patient-derived PDAC tissues (**Figure 1E-G**) and NSCLC tissues (**Figure 1H-J**) across various cancer stages. Additionally, S15 expression was confirmed in multiple human and mouse cancer cell lines (**Figure S1B&E**), tumor-associated macrophages (**Figure 1F**, middle panel), and tumor-associated stromal cells (**Figure S1F**, right panel). We compared the expression of S15 on CD19+ B cells, CD3+ T cells and CD14+ monocyte cell populations within human PBMCs relative to H460 NSCLC and Panc-1 PDAC cancer cells, and found detectable but markedly lower S15 expression relative to the cancer cells (**Figure S2**).

### Design and characterization of STAB

**Figure 2:**
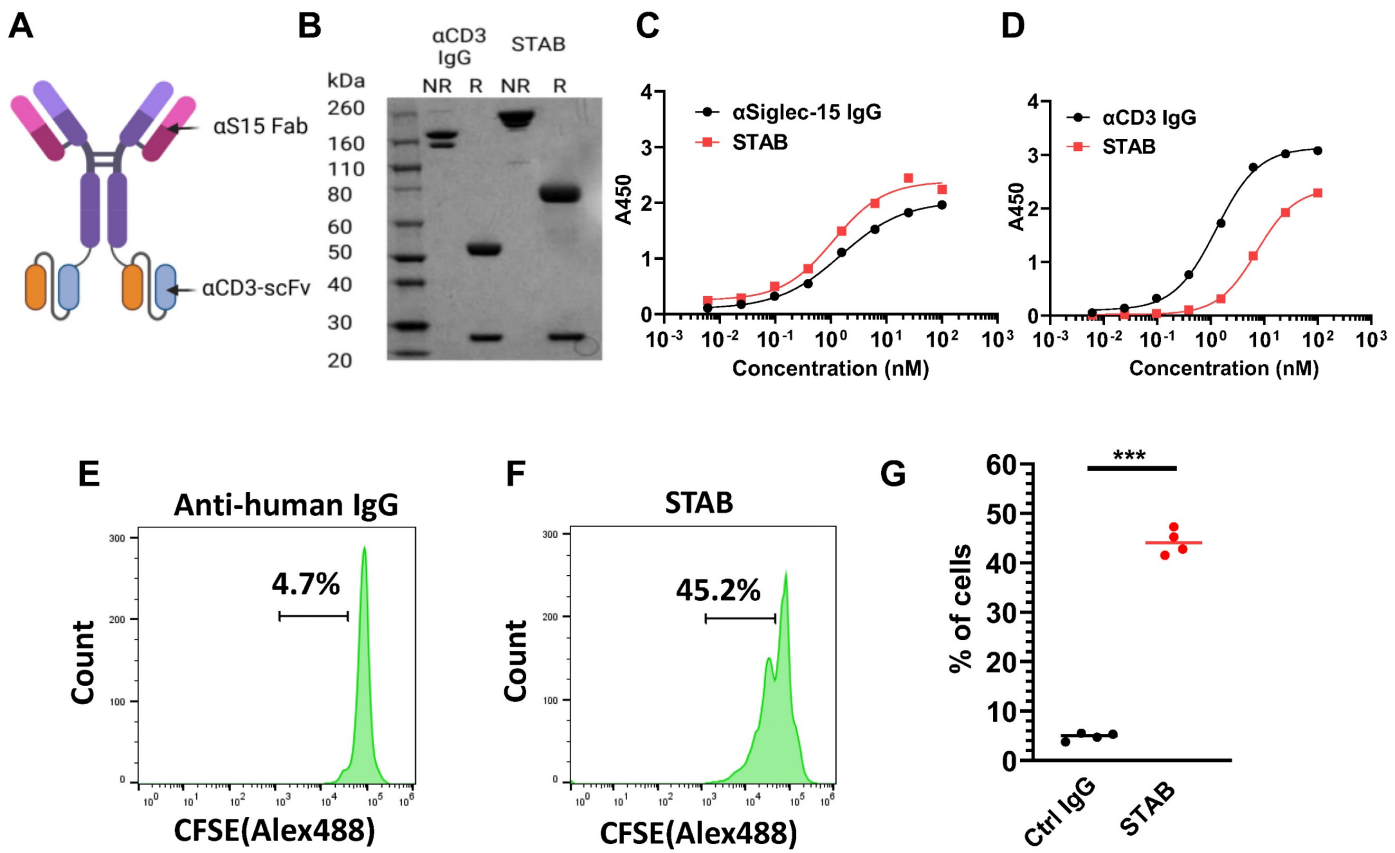
** Characterization of STAB *in vitro*. (A)** Schematic representation of the STAB molecule in the IgG-scFv format that binds CD3 and S15. **(B)** SDS-PAGE gel analysis confirms the expression of the STAB construct, showing correctly sized heavy and light chains. **(C)** Binding affinity of STAB to S15 compared to the parent S15 mAb, measured by ELISA. **(D)** Binding affinity of STAB to CD3 compared to the parent CD3 mAb, measured by ELISA. **(E-F)** Representative flow cytometry histograms demonstrating proliferation of T cells induced by **(E)** control anti-human IgG and **(F)** STAB, assessed using the CFSE proliferation assay. PBMCs from different donors were stained with 5 μg CFSE. After staining, PBMCs were cultured with either control IgG or STAB for 3 days, followed by harvesting and preparation of single-cell suspensions, and finally analysis by flow cytometry. **(G)** Quantification of fraction of CD3⁺ T cells following control IgG or STAB treatment. Data are presented as the mean ± standard error of the mean (SEM). ***p < 0.001.

We engineered the bispecific STAB molecule in the popular IgG-scFv format (**Figure 2A**). Each molecule consists of symmetric heavy chains dimerized via human IgG1-Fc, resulting in 2 Fabs against S15 [17]and 2 scFv's against CD3 adapted from the popular anti-CD3 mAb UCHT1 18, linked via a 15 amino acid flexible glycine-serine linker to the C-terminus of the Fc. Under non-reducing conditions, we observed a primary band at the molecular weight corresponding to the theoretical size of an intact STAB (**Figure 2B**), whereas under reducing conditions we observed two bands corresponding to the theoretical size of the heavy (78kDa) and light chains (25kDa). We next assessed the binding affinities of STAB to S15 and CD3 via ELISA. The EC_50_ of STAB against S15 is ~1.1nM, which is highly comparable to the ~1.5nM EC_50_ found with the parent IgG (**Figure 2C**). The binding affinity of STAB against CD3 (EC_50_ ~7.3nM) was slightly reduced compared to the parent IgG (EC_50_ ~1.3nM), but remained in the single-digit nanomolar range reflective of strong affinity to CD3 (**Figure 2D**).

To further confirm CD3 binding on T cells, we incubated STAB or control IgG with T cells in PBMCs previously stained with carboxyfluorescein (CFSE) diacetate for 72 hs, and analyzed them using flow cytometry. Compared to a non-specific IgG control, STAB induced a significantly higher abundance of T cells within PBMCs, increasing the fraction of proliferated T cells in 4 of 4 unique donors sampled by nearly 10-fold from ~4.7% in PBMCs treated with control IgG to ~45% in PBMCs from three different donors (**Figure 2E-G**). In contrast, STAB did not increase the activation of myeloid cells, as the expression of MHCII and CD80 on CD14+ monocytes remained low (**Figures S3**). These results not only provide independent confirmation that STAB can effectively bind T cells, but also suggest that STAB can significantly enhance the proliferation of T cells through CD3-mediated stimulation.

### STAB promotes efficient killing of S15^+^ PDAC and NSCLC cells in co-culture studies

**Figure 3:**
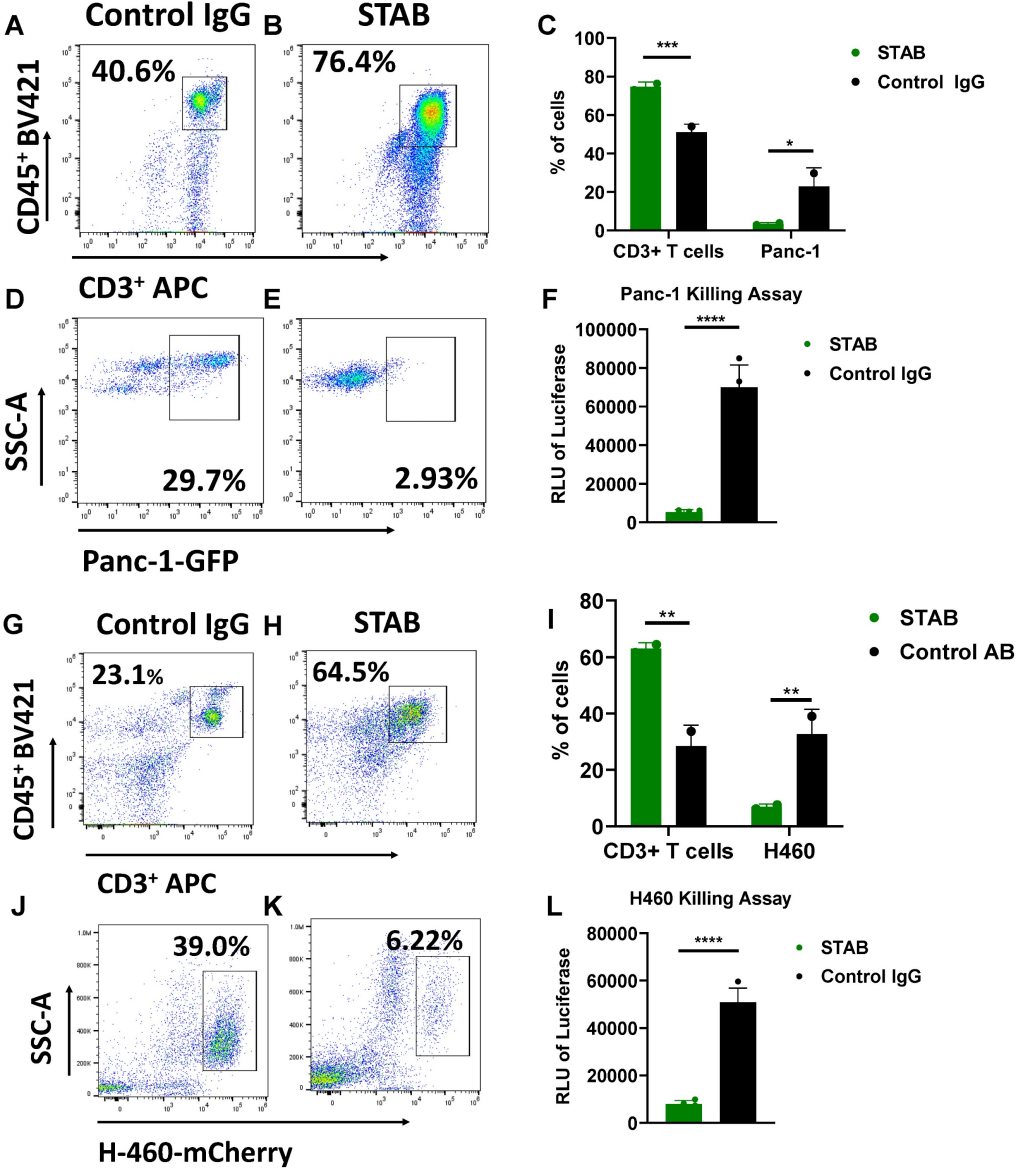
** STAB enhances T cell-mediated killing of S15+ human Panc-1 PDAC cells and human H460 NSCLC cells *in vitro*.** PBMCs from different donors were stained with 5 μg CFSE, then cultured with tumor cells in presence of either Control IgG or STAB for 3 days, followed by harvesting and preparation of single-cell suspensions. CD3+CD45+ cell and tumor cell populations were analyzed by flow cytometry (n = 4). **(A-B)** Representative flow cytometry gating strategy for CD3⁺CD45⁺ T cells in Panc-1 co-culture. **(C)** Quantification of CD3⁺ T cells and GFP⁺ Panc-1 cells after treatment with Control IgG or STAB. **(D-E)** Representative flow cytometry gating strategy for GFP⁺ Panc-1 cells. **(F)** Luminescence-based assay results from Panc-1 co-culture with PBMCs treated with STAB or Control IgG, showing STAB-mediated tumor cell killing. **(G-H)** Representative flow cytometry gating strategy for CD3⁺CD45+ T cells in H460 co-culture. **(I)** Quantification of CD3⁺ T cells and mCherry⁺ H460 cells after treatment with Control IgG or STAB. **(J-K)** Representative flow cytometry gating strategy for mCherry⁺ H460 cells. **(L)** Luminescence-based assay results from H460 co-culture with PBMCs treated with STAB or Control IgG, showing STAB-mediated tumor cell killing. Percentage of cells in the STAB-treatment group is presented relative to the Control Ab at an effector-to-target (E:T) ratio of 3:1. Statistical differences in cell killing between STAB and control IgG were assessed using two-way ANOVA and are denoted as * (*p < 0.05; **p < 0.01).

We next investigated whether STAB can enhance T cell mediated killing of specific cancer cells *in vitro*. We mixed Panc-1 PDAC expressing GFP with human PBMCS at a ratio of 3 PBMCS to 1 cancer cell (E:T 3:1), followed by addition of either STAB or control IgG. In Panc-1/PBMC co-cultures treated with control IgG, there was a substantial cancer cell population (~23 ± 6%) consistent with a lack of cancer cell killing (**Figure 3D**). In contrast, addition of STAB reduced the Panc-1 population to less than ~3 ± 0.8% (**Figure 3E**, p < 0.05). The marked reduction of cancer cells is correlated with greater abundance of T cells, from ~46 ± 5.5% of all cells in control IgG group (Figure 3 A) to ~75 ± 1.4% in the STAB treatment group (p < 0.01; **Figure 3B**). We also verified STAB's anti-tumor activity against H460 NSCLC cells expressing mCherry. Similar to PDAC, the addition of STAB to H460:PBMC co-cultures markedly reduced the fraction of H460 cells from ~37 ± 5% to ~7 ± 0.7% (p < 0.01; **Figure 3J, K**), with a concurrent increase in the fraction of T cells from ~28 ± 5% to 63 ± 2% (p < 0.05; **Figure 3G, H**). To further validate the flow cytometry data, we employed a luciferase-based assay as an independent marker for tumor cell activity. As shown in **Figure 3F** and **3L**, STAB treatment induced significantly higher tumor cell killing compared to control IgG. STAB mediated killing is concentration dependent, with greater STAB concentration leading to more effective elimination of Panc-1 and H460 cancer cells (**Figure S4**). To verify that it is indeed T cells within PBMCs that mediated the killing, we performed additional studies where Panc-1 and H460 are co-cultured with either PBMC or purified CD3+ T cells. We observed comparable killing of both cancer cells by CD3+ T cells (**Figure S5**), indicating that the STAB-induced cytotoxicity in the presence of PBMCs is indeed mediated by CD3+ T cells.

While S15 is overexpressed on many cancers, the degree of S15 overexpression varies. To assess the degree to which variable S15 expression may impact the effectiveness of STAB-redirected killing by T cells, we performed additional co-culture studies with cell lines with lower S15 expression. Not surprisingly, in *in vitro* co-culture assays, we observed STAB-induced killing was dependent on the expression levels of S15 on tumor cells. These results further validate the specificity and efficacy of our approach and highlight its dependence on Siglec-15 expression levels (**Figure S6**). Altogether, these results underscore the ability for STAB to activate T cells, induce T cell proliferation, and redirect T cells to kill S15+ cancer cells.

### STAB inhibition of established human pancreatic tumors correlates with increased T cell activation and reduced stromal barrier

**Figure 4:**
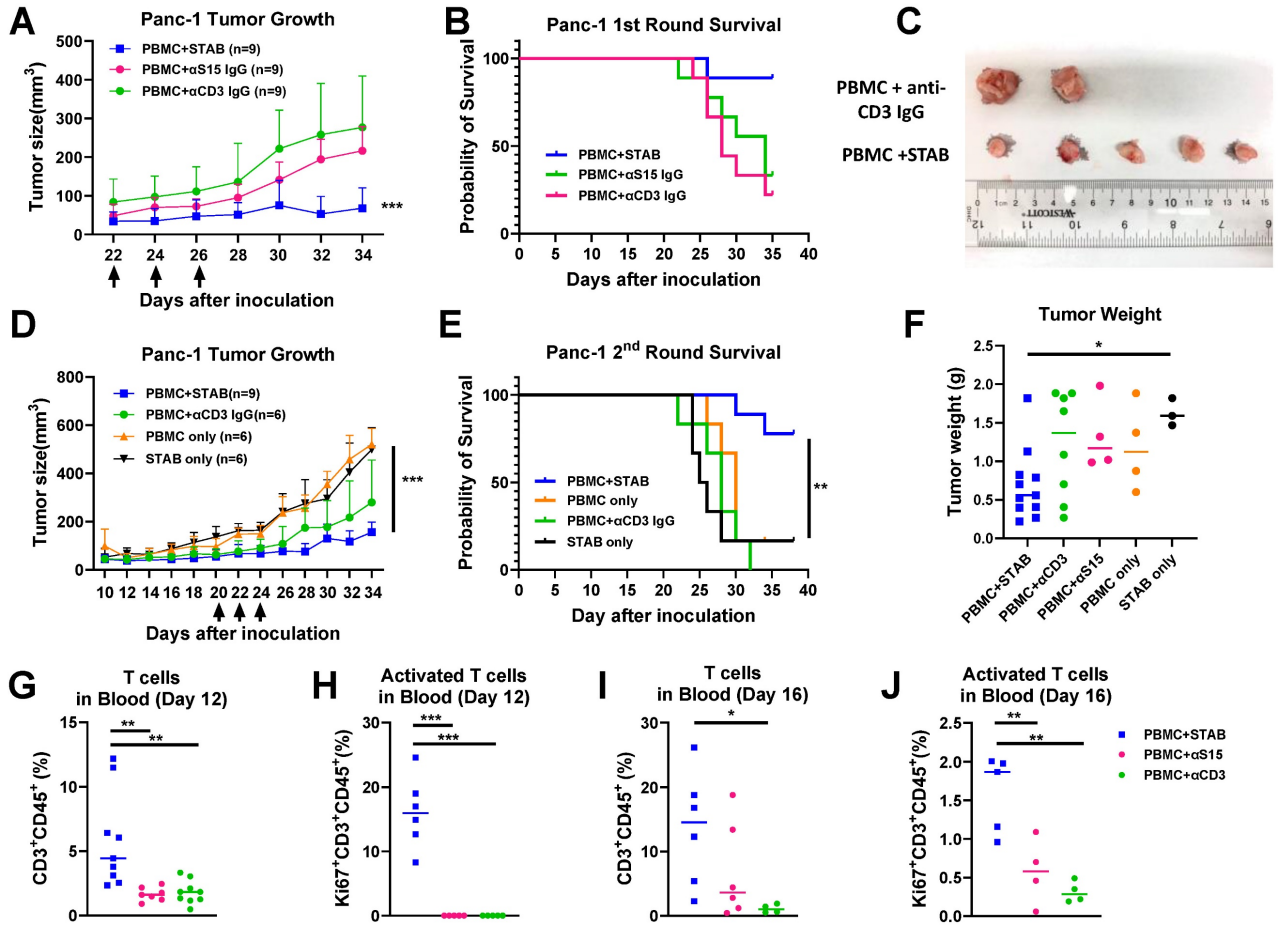
** STAB effectively inhibits Panc-1 tumor growth and significantly prolongs survival compared to treatments with anti-S15 or anti-CD3 mAbs. (A&D)** Inhibition of Panc-1 tumor growth in NSG mice. Panc-1 cells (1 × 10⁶ in 50 μL PBS) were injected subcutaneously, and tumor size was measured every 2 days using calipers. Panels A and D represent two independent experiments (1st round: n = 5; 2nd round: n = 6-9). **(B&E)** Kaplan-Meier survival curves for the mice in **(A) & (D)**. Survival was defined by humane endpoints or tumor size exceeding 1000 mm³. Each group comprised 6-9 mice. **(C)** Representative images of tumors from the control and STAB treatment groups at the study endpoint. **(F)** Tumor weights at the study endpoint. **(G-J)** Quantification of CD3⁺ T cells and activated Ki67⁺CD3⁺ T cells in peripheral blood at 12 and 16 days post-treatment. Blood (60 μL) was collected via submandibular bleeding, stained with CD3, CD45, and Ki67, and analyzed by flow cytometry. Statistical differences between STAB treatment and control anti-human IgG groups were assessed using one-way ANOVA. Results are denoted as * (*p < 0.05; **p < 0.01; ***p < 0.001).

Based on these promising results *in vitro*, we proceeded to evaluate the anti-tumor efficacy of STAB in a human PDAC model in NSG mice. Once the Panc-1 tumor volume had reached ~150-200mm^3^, human PBMCs from healthy donors as well as either STAB or control IgGs were infused a total of 3 times over a 5-day period into Panc-1 tumor-bearing mice During the treatment period, all mice survived, and there was no significant difference in body weight between STAB treatment group *vs.* control IgGs. STAB effectively inhibited the growth of PDAC tumors compared to mice receiving either anti-S15 or anti-CD3 mAb (**Figure 4A**), which resulted in prolonged survival of STAB-treated mice (**Figure 4B**). To confirm the observed efficacy, we executed a second independent study, and again observed far superior tumor suppression and prolonged survival in mice treated with PBMC and STAB *vs.* either STAB alone or other PBMC negative controls (**Figure 4D, E**). STAB-mediated tumor suppression is also confirmed by the difference in tumor weights and tumor size at the study endpoint (**Figure 4C, F**).

The strong efficacy we observed motivated us to seek further insights into the anti-tumor activity of STAB. We analyzed blood collected from the animals on Days 12 and 16 post last treatment (i.e. Day 36/38 and day 40-42 post tumor-inoculation). In good agreement with our *in vitro* findings, by Day 12, STAB significantly increased the fraction of circulating CD3+CD45+ T cells by ~7-fold and ~4-fold over those in mice given anti-S15 IgG and anti-CD3 IgG, respectively (**Figure 4G**). Importantly, STAB treatment increased the fraction of activated T cells (i.e. Ki67^+^) by ~3.3-fold and ~4.3-fold compared to mice given anti-S15 IgG and anti-CD3 IgG, respectively (**Figure 4H**). This trend continued through Day 16, with the abundance of CD3+CD45+ T cells in STAB-treated mice remaining ~2 fold and ~12-fold greater than those in mice given anti-S15 IgG and anti-CD3 IgG, respectively (**Figure 4I**). The abundance of activated T cells in STAB-treated mice were appreciably lower than what was measured on Day 16, but remained ~2.83-fold and ~5-fold over those in mice given anti-S15 IgG and anti-CD3 IgG, respectively (**Figure 4J**).

**Figure 5:**
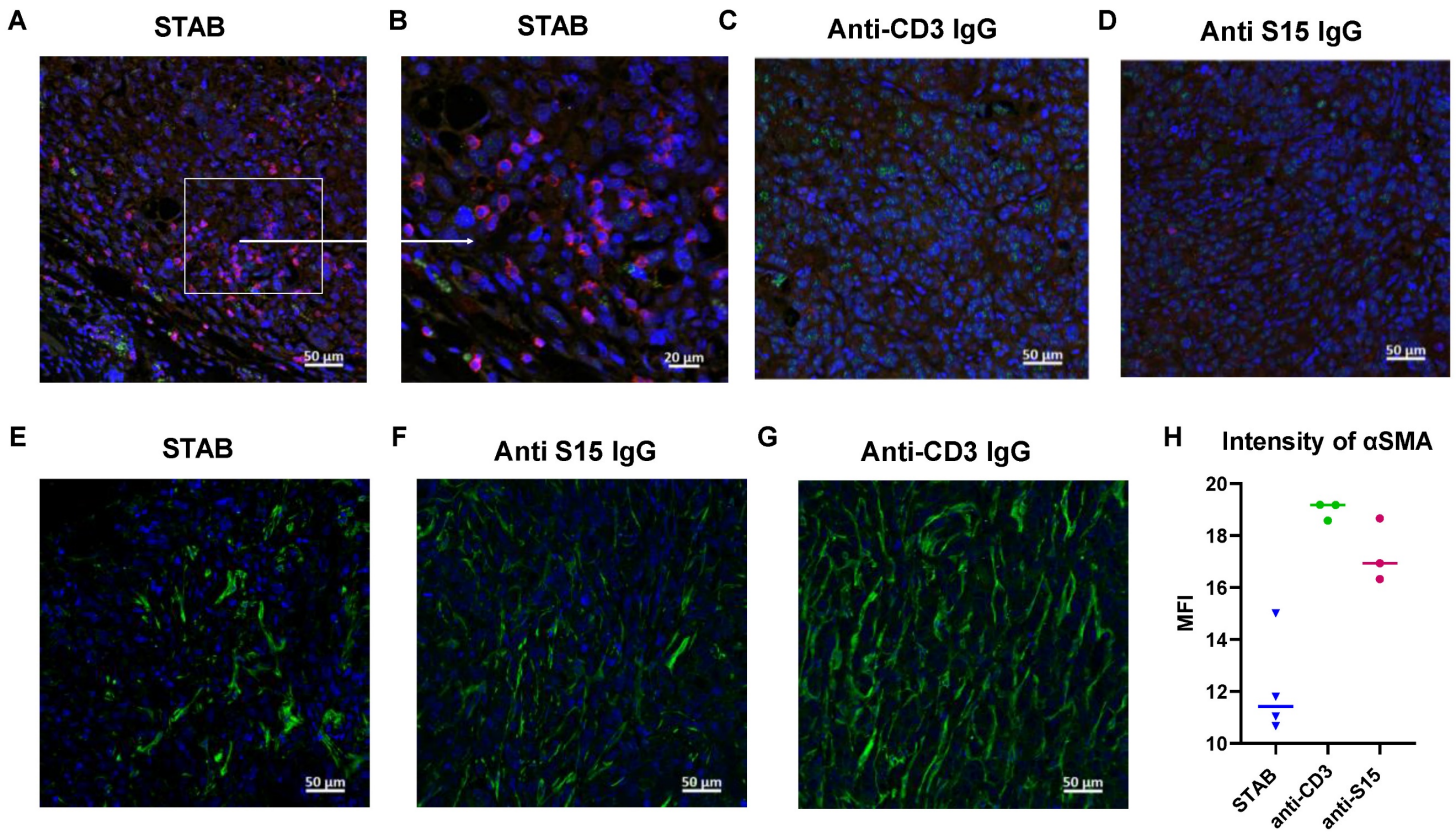
** STAB treatment increases T cell infiltration in tumors and reduces tumor-associated fibroblasts in desmoplastic Panc-1 tumors.** Tumors were harvested at the study endpoint from different treatment groups, fixed in 10% formalin overnight, and then stored in 70% ethanol before being embedded in paraffin. **(A-D)** Tumor sections were stained with anti-CD3 (Alexa 594, red) and anti-S15 (Alexa 488, green) to evaluate T cell infiltration and S15 expression. Negative staining (without primary antibody or without secondary antibody) and control staining were performed. **(E-G)** Tumor sections were stained with anti-αSMA (Alexa 488, green) and DAPI (blue) to assess tumor-associated fibroblasts. Negative staining and control staining were also performed. **(H)** Bar graph showing quantification of αSMA staining as an indicator of fibroblast presence.

Given the increase in abundance of T cells in the circulation, including activated T cells, we next assessed whether STAB also enhanced T cell distribution into and retention in the tumors. We performed immunohistochemistry on tumor sections, and found appreciably greater abundance of CD3+ T cells infiltrate within the tumors of mice treated with STAB compared to control groups (**Figure 5A-D**). Additionally, we observed that STAB treatment increases IFN-γ production by T cells, indicating enhanced cytotoxic activity (**Figure S7**). Altogether, these results suggest the superior anti-tumor activity afforded by STAB is likely a consequence of enhanced intratumoral as well as systemic T cell activity.

A hallmark of PDAC, and what makes PDAC such a difficult cancer to treat is the abundance of tumor-associated fibroblasts (TAFs). TAFs not only can serve as a source of pro-tumorigenic signals, but also creates a stromal barrier that strongly limits the tumoral distribution of various therapeutics. Interestingly, STAB appears to markedly reduce the stroma barrier present in the PDAC tumors (**Figure 5E-H**), which may in turn enhanced the infiltration of circulating immune cells as well as STAB into the tumor. The reduced TAF may be caused by STAB redirection of circulating T cells to TAF, which is consistent with our unpublished observations that TAF also harbors elevated levels of S15.

### STAB also inhibits NSCLC tumors *in vivo* and prolonged T cell circulation

**Figure 6:**
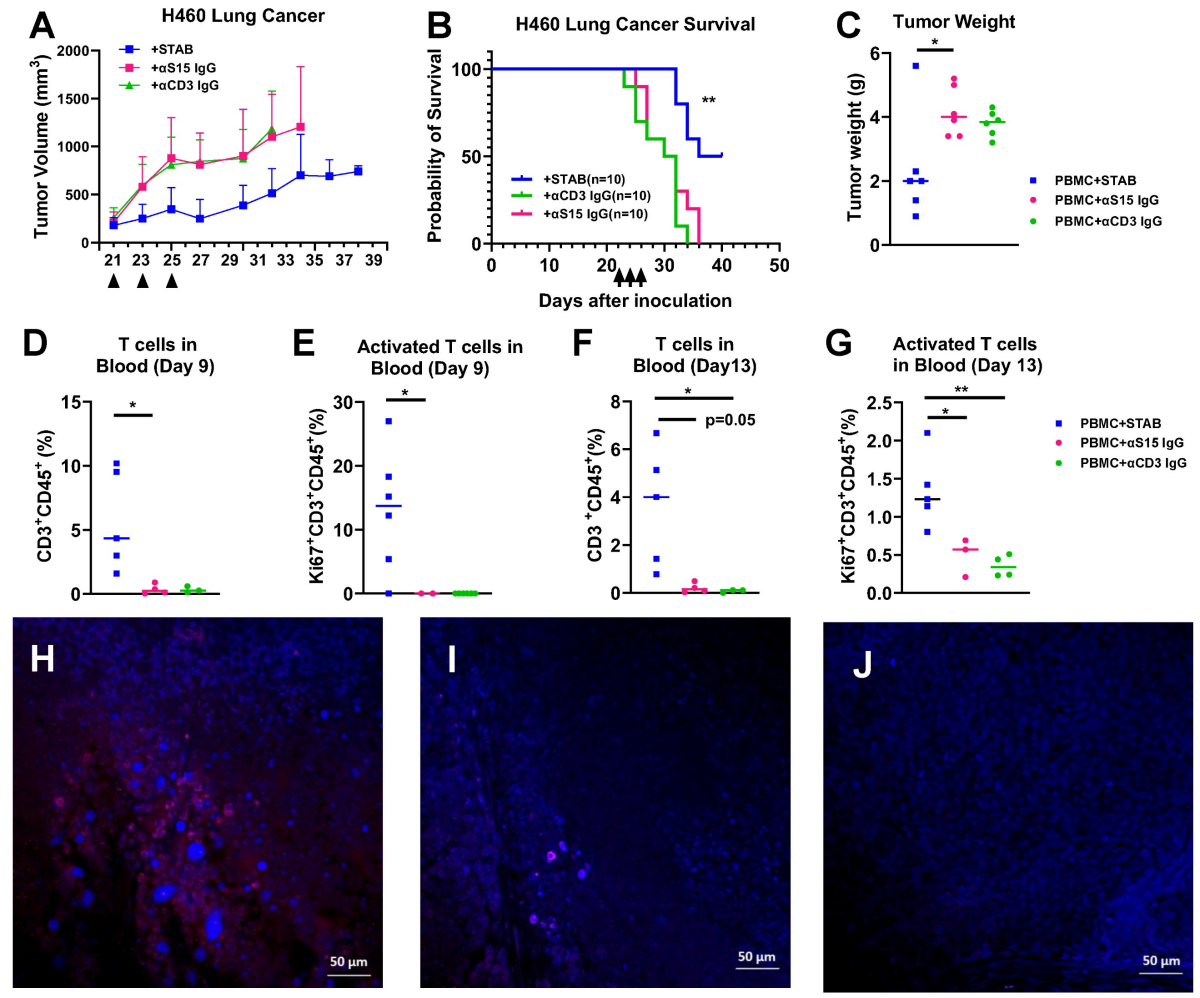
** STAB treatment inhibited tumor growth and increased T cell infiltration in H460 NSCLC tumor. (A)** Tumor inhibition and (B) Kaplan-Meier plot for survival after STAB and control Abs treatment in H460 lung tumor models in NSG mice. H460 cells (1 × 10⁶ in 50 μL PBS) were injected subcutaneously, and tumor growth was measured every 2 days using calipers. Survival was defined by humane endpoints or tumor size exceeding 1000 mm³. Panels A, B and C represent data from two independent experiments (1st round: n = 5; 2nd round: n = 5). **(C)** Tumor weights at the study endpoint. **(D-G)** Quantification of CD3⁺ T cells and activated Ki67⁺CD3⁺ T cells in peripheral blood at 9 and 13 days post-last treatment. Blood (60 μL) was collected via submandibular bleeding, stained with CD3, CD45, and Ki67, and analyzed by flow cytometry. **(H-J)** Tumor were harvested at the end of the study for histology. The sections were stained by CD3+ Alex 594 in red and DAPI in blue. Negative staining and control staining were performed. Statistical differences between STAB treatment and control anti-human IgG groups were assessed using one-way ANOVA. Results are denoted as * (*p < 0.05; **p < 0.01).

We further evaluated the efficacy of similar STAB treatment in H460 NSCLC model in NSG mice. Similar to our findings in mice with PDAC tumors, STAB treatment again strongly limited the growth of H460 tumor over time (**Figure 6A**), which translated to markedly prolonged overall survival (**Figure 6B**). The tumor weights in STAB-treated group were significantly lower than the control groups (**Figure 6C**). The anti-tumor activity observed correlated with elevated levels of number of circulating CD3^+^ T and activated (Ki67^+^) CD3^+^ T cells in the blood 9 day and 13 days after last treatment (**Figure 6D-F**). We similarly found increased T cell infiltration in the STAB treatment group (**Figure 6G**) compared with groups treated with control mAbs (**Figure 6H, I , J**).

### Toxicity evaluation

Finally, we evaluated the potential toxicity of the STAB treatment via toxicological pathology analysis. The serum biochemical parameter analysis and the whole blood cell counts remained within the normal ranges for all the groups, showing no systemic anemia or inflammation induced after treatments and no major liver disfunctions (**Table S1**).

## Discussion

Due to the difficulty in early diagnosis, PDAC frequently presents as unresectable advanced stage disease, with standard of care such as chemotherapy and/or radiotherapy improving survival at best by only several months 19. Despite the promise of immunotherapy across a spectrum of cancer, its clinical benefit for PDAC remains limited 20, likely due to the highly immunosuppressive TME as well as the surrounding desmoplastic stroma that severely limits access of immune cells and immunomodulators. Indeed, PDAC is characterized by a dense and rich stroma, composed of immunosuppressive cells, fibroblasts and various cell types 21, with interactions between stroma and cancer cells contributing to both tumor proliferation and resistance to therapies. To address the clinically unmet need for better therapies for PDAC, in addition to facilitate direct killing of the cancer cells, we believe we must simultaneously break down the stroma barrier and alleviate the immunosuppressive TME. To achieve all 3 aims with a single intervention, we took advantage of the fact that S15, which possess immunosuppressive functions similar to PD-L1, is strongly upregulated on the surface of both primary PDAC and surrounding stroma. This allows us to utilize the bispecific STAB molecule to redirect T cells to bind and kill both types of S15+ cells, with S15 binding in the tumor likely further reducing potential immunosuppression within the TME. In good agreement with our hypothesis, we found STAB markedly reduced the stromal barrier and facilitate effective killing of PDAC, leading to marked suppression of tumor growth and prolonged survival* in vivo*. These results underscore S15 as a promising immunotherapy as well as immune cell therapy target for PDAC.

Beyond PDAC, we showed STAB also offered considerable efficacy in suppressing NSCLC growth *in vivo*, another aggressive tumor with high mortality rate that strongly overexpresses S15, particularly in lung adenocarcinoma 9. Our work adds to an increasing array of studies that support the emergence of S15 as an attractive tumor biomarker and target for immunotherapy 22. Indeed, S15 is broadly present on many other cancers, including triple negative breast cancer (TNBC), head and neck cancer, kidney cancer, colon cancer, and more 23-25. Our findings here that STAB offered considerable efficacy against both PDAC and NSCLC, coupled with our recent findings that a bsAb against S15 and TGF-b was able to meaningfully attenuate the immunosuppressive TME in TNBC 25, 26, suggests STAB may offer similar efficacy for TNBC as well as a wide hosts of other S15+ tumors.

The *in vivo* efficacy of STAB is most certainly multi-faceted. First and foremost, STAB binding to CD3ε on T cells appear highly effective at redirecting T cells to bind and kill S15+ cancer cells and fibroblasts, as illustrated by our *in vitro* and *in vivo* studies. Beyond facilitating specific T cell killing, STAB also appeared to directly stimulate T cells, contributing to appreciably greater abundance of T cells in the circulation. Indeed, we detected substantial number of activated T cells in the circulation as late as 12 and 16 days after the last STAB infusion. In turn, this likely enhances the number of T cells available that could extravasate and reduce the desmoplastic tumor structure in PDAC, leading ultimately to greater infiltration into tumors and more effective anti-tumor response. Consistent with this mechanism, we saw appreciably greater activated T cells within the tumors. Third, S15 is associated with tumor immune evasion, including regulating macrophage activation and functions of tumor-associated macrophages. Wang *et al*. showed that S15 inhibited T-cell growth and activation. Thus, our use of the same anti-S15 Fab previously used to inhibit S15 activity should also contribute to attenuating the immunosuppressive TME in PDAC and NSCLC tumors. Interestingly, the expression patterns of S15 and PD-1 appeared to be mutually exclusive in diverse cancers including PDAC, lung, TNBC, head and neck squamous, and bladder cancers; thus, in patients with such cancers that are PD-1 negative, S15 may play a major role in the observed immunosuppression. This suggests STAB may be particularly effective in patients who do not react to anti-PD-1/PD-L1 medication 9.

STAB possess many of the same advantages of the conventional biTE. Unlike patient-derived T cells modified exogeneously to express select chimeric antigen receptor (CAR-T) followed by reinfusion, STAB would be available off the shelf and can be readily manufactured in large quantities. This directly improves access, allows repeated dosing, and greatly lowers potential costs. Unlike CAR-T that requires a 6-8 weeks delay, a delay during which as many as ~30% of the patients would deteriorate and become ineligible for the therapy or deceased, the immediate initiation of treatment afforded by STAB should directly increase the number of patients who can benefit from the treatment. Furthermore, unlike conventional CAR-T, no lymphodepletion is necessary, and there is no risk of modified T cells becoming cancerous 27-29. These distinguishing features should afford better quality of life to the patients, both in the short term and in the long run. Nonetheless, STAB-based therapy, similar to other T cell engager molecules, is only feasible in patients with sufficient peripheral immune cell counts; this is often limited by pre-existing T cells landscape 30, 31 More importantly, STAB alone was only able to suppress tumor growth, and was unable to eradicate the tumor.

BiTE and other forms of T cell engagers have been heavily explored for solid tumor like PDAC and NSCLC 32, 33. For instance, EGFR-CD3 BiTE 34-36 armed activated T cells could enhance subsequent responsiveness to chemotherapeutic drugs, but the impact of the BiTE in pancreatic tumor microenvironment on the activity and infiltration of T cells remains to be fully understood. Mesothelin-CD3 BiTE redirects T cells to this highly overexpressed antigen on PDAC and NSCLC 34, but does not mitigate the immunosuppressive TME nor target TAF. Overall, we consider a relatively distinguishing feature of our STAB approach is the multiple mechanisms of action, including redirecting T cells to kill not just the primary tumor but also tumor associated M2 macrophages and stromal cells, as well as alleviate immunosuppressive TME. The breakdown of the stroma barrier likely enhances the T cell penetration into the tumor, with the consequent killing of the M2 macrophages likely supports more effective killing of cancer cells. We draw inspiration from Tarlatamab's, a recently approved DLL3-CD3 BiTE that enables multiple mechanism of protection by targeting the atypical DLL3 Notch ligand whose overexpression promotes the growth of small cell lung cancer and also enhances their migratory and invasive capacity 37.

Compared to other checkpoint inhibitors such as PD-1/PDL-1, S15 continues to be a rarely exploited target for immunotherapies. This is likely attributed to the lack of clinical efficacy of NC-318 9, a monotherapy based on an anti-S15 mAb, in a Phase 1/2 study of patients across a multitude of advanced solid tumors including lung, breast, endometrial tumors 38. The lack of responders in the clinical study raised questions about the clinical utility of S15 targeting. In our study, the inability of STAB to fully eradicate PDAC and NSCLC may be construed as support for the futility of S15-targeting. We were not surprised by the limited efficacy of NC318, given its monotherapy nature. The molecular pathways conferring an immunosuppressive TME is inherently complex, with multiple distinct contributing pathways 39-41. Blockade of any single pathway is unlikely to be sufficient in most patients. Indeed, even the wildly successful PD-1/PD-L1 axis is only efficacious in ~10-20% of patients with various solid tumors when utilized as a monotherapy target. Rather than serving strictly as an immunotherapy target, we see S15 as a promising tumor and stroma binding target, with the added benefit of partial reducing the immunosuppressive TME. S15 shows distinctive expression in tumor tissues, including in M2 macrophages, and generally limited expression in normal tissues 9. Compared to other well-established cancer targets such as B7-H3 42, 43, EGFR 44-46 and MUC-1 47, S15 has rarely been exploited as an immune cell therapy target. When combined with suitable immune checkpoint inhibitors, we believe STAB could be part of an effective immune cell therapy.

## Materials and Methods

### Bispecific antibody design and production

The DNA was synthesized through Twist Biosciences and cloned into mammalian expression vector pαH. The heavy and light chain DNA was transfected into Expi293 cells in a 1:1 ratio using manufacturer protocols using expifectamine as the transfection reagent. (Thermofisher). The cell culture supernatant was collected once cell viability dropped below 60% and was purified using Protein-A/G affinity chromatography.

### Sodium dodecyl-sulfate polyacrylamide gel electrophoresis (SDS-PAGE)

The prepared IgG-scFv was analyzed by SDS-PAGE with Imperial Protein Stain. To verify the molecular weight, non-reduced and reduced SDS-PAGE were performed. The non-reduced sample was prepared by mixing 5 µg of the protein with 5 µl 4x NuPage^TM^ LDS Sample Buffer and diluting to 20 µl. Reduced samples were prepared similarly, with the addition of 1 µl TCEP as a reducing agent. Samples were then incubated for 10min at 70°C. The sample was separated using a 4-12% NuPage^TM^ Bis-Tris gel. The SDS-PAGE gel was stained for 1hr with Imperial Protein Stain and decolorized overnight before image capture with the ChemiDoc MP Imaging system (Bio-Rad).

### Enzyme-linked immunosorbent assay (ELISA)

Either human CD3ε protein (Novus Biologicals, Cat # NBP2-22752) or human S15 protein (Acro Biosystems, Cat # SG5-H52H3), were coated as antigen onto high binding, half-area, clear 96-well plates (Corning Costar, Cat # 3690) overnight at 4˚C. Proteins were plated at 50 µl/well, 2 μg/mL in carb-bicarb buffer (pH 9.6, Sigma C3041) for overnight coating. The next morning, plates were washed 5x with PBS-0.05% Tween (PBST) and subsequently blocked for 1 h at room temperature with 150 µl/well 5% w/v non-fat milk in PBST. Following 3x PBST washes, purified antibody samples and controls were serially diluted in 1% w/v milk-PBST, added to the blocked plates at 50 µl/well for 1 h incubation at room temperature. Plates were washed 3x with PBST, and bound antibodies were detected using 50 µl/well of F(ab')2-Goat anti-Human IgG Fc Cross-Adsorbed Secondary Antibody, HRP (Invitrogen, Cat# A24476) at 1:10,000 dilution in 1% w/v milk-PBST incubated for 1 h at room temperature. Following 3x PBST washes to remove unbound secondary detection antibody, 50 µl/well 1-Step Ultra TMB-ELISA substrate solution (Thermo Scientific) was added for up to 10 mins to detect HRP activity. The enzymatic reaction was quenched by adding equal volume of 1 N HCl, and the color development was immediately determined by taking absorbance measurements at 450 nm (signal) and 570 nm (background) wavelengths using a SpectraMax M2 microplate reader (Molecule Devices). Negative control wells, including antigen coated, blocked wells without primary antibody incubation and uncoated, blocked wells with primary antibody incubation, both revealed negligible signal development in the assay. Background subtracted absorbance values for each sample condition, run in triplicate, were imported into GraphPad Prism 8 software. EC50 was calculated using a Sigmoidal 4PL non-linear curve fit.

### Cell line and cell culture

The human pancreatic cancer cell line Panc-1 was obtained from the Tissue Culture Facility at the University of North Carolina (UNC), and the human non-small cell lung cancer (NSCLC) cell line H460 was kindly provided by Dr. Hingtgen's laboratory. Panc-1 cells were cultured in Dulbecco's Modified Eagle Medium (DMEM), while H460 cells were maintained in RPMI-1640 medium. Both culture media were supplemented with 10% fetal bovine serum (FBS; Gibco) and 1% Penicillin-Streptomycin (Pen/Strep; Gibco) to ensure optimal cell growth and prevent bacterial contamination. Cells were cultured in a humidified incubator at 37°C with 5% CO₂. Cell cultures were regularly monitored for morphology, confluency, and contamination, and media were replaced every 2-3 days. Sub-culturing was performed when cells reached approximately 80-90% confluence using 0.25% Trypsin-EDTA (Gibco) for detachment.

### Human peripheral blood mononuclear cells (PBMCs) isolation from Buffy coat

For *in vitro* studies, human PBMCs were isolated from buffy coats obtained from the American Red Cross. The buffy coat was first diluted at a 1:2 ratio with phosphate-buffered saline (PBS; without Ca²⁺/Mg²⁺, room temperature) and gently mixed to ensure homogeneity. Next, 20 mL of the diluted cell suspension was carefully layered over 15 mL of Ficoll-Paque density gradient medium in a 50 mL conical tube. The samples were centrifuged at 300 × g for 30 mins at room temperature without braking to maintain the gradient separation. Following centrifugation, the upper plasma layer was aspirated, taking care to leave the mononuclear cell layer undisturbed at the interphase. The mononuclear cell layer (containing lymphocytes, monocytes, and thrombocytes) was carefully transferred to a new 50 mL conical tube. To remove residual Ficoll and plasma, the cells were washed by adding PBS supplemented with 1 % fetal bovine serum (FBS) and centrifuged at 350 × g for 10 mins at 4°C. After centrifugation, the supernatant was carefully removed, and the resulting cell pellet was resuspended in PBMC culture medium. The isolated PBMCs were then prepared for further *in vitro* analysis and experimental studies.

### Thawing and preparing cryopreserved PBMCs

For *in vivo* tumor studies, frozen PBMCs were purchased from STEMCELL Technologies. These PBMCs were thawed and processed according to the manufacturer's instructions prior to use. Frozen PBMCs (10⁷-50⁷ cells per vial) are thawed rapidly in a 37 °C water bath until a small ice crystal remains. Pre-warmed culture medium (1 mL, 37 °C) is then added to the cryotube, and the cells are transferred to a 50 mL tube, rinsing the cryotube with an additional 1 mL of medium. The 50 mL tube is then filled with culture medium and centrifuged at 300 × g for 10 mins. The supernatant is discarded, and the cell pellet is gently resuspended in 1 mL of fresh medium, then diluted to a total volume of 50 mL. The cells are incubated in a CO₂ incubator for 2-4 h with the cap slightly open to allow recovery. After resting, the cell suspension is centrifuged at 300 × g for 10 mins at room temperature and resuspended in 20 mL of culture medium. Finally, the cells are counted, viability is assessed, and the concentration is adjusted as needed for downstream applications.

### *In vitro* T-cells proliferation assay

T cell proliferation in this study was assessed using the carboxyfluorescein succinimidyl ester (CFSE) fluorescent dye to monitor cell division. PBMCs were first isolated from buffy coats as described previously and labeled using the CellTrace™ CFSE Cell Proliferation Kit (Cat# C34554, ThermoFisher®).

The CFSE working solution was prepared by diluting the stock solution in 20 mL of pre-warmed PBS (37°C) to a final concentration of 5 µM. A cell suspension of 1 × 10⁶ cells/mL (10 mL total volume) was centrifuged at 300 × g for 5 mins at 4°C. The supernatant was carefully aspirated, and the cell pellet was resuspended in 10 mL of the CFSE staining solution. Cells were incubated for 20 mins in a 37°C water bath to allow CFSE uptake. To quench unbound CFSE, 40 mL of pre-warmed PBMC medium was added to the cells, followed by incubation at 37°C for 5 mins. The cells were then centrifuged at 300 × g for 5 mins, and the supernatant was carefully removed. The resulting cell pellet was resuspended in pre-warmed PBMC medium. For the proliferation assay, 5 µg of STAB was added to 500 µL of cells (1 × 10⁶ cells/mL) in a 24-well plate. The cells were cultured for 72 hs at 37°C in a humidified incubator with 5% CO₂. After the incubation period, T cell proliferation was analyzed by flow cytometry, with CFSE dye dilution serving as an indicator of cell division and activation.

### *In vitro* co-culture killing assay

We assessed the ability of STAB to facilitate T cells mediated elimination of Panc-1 and H460 cancer cells. Panc-1 cells stably expressing GFP and H460 cells stably expressing mCherry were seeded into 24-well flat-bottomed plates (0.5 × 10⁵ cells/well) and allowed to adhere overnight. After 24 hs, the culture medium was replaced with fresh DMEM (for Panc-1) or RPMI-1640 (for H460) containing 5 µg of STAB. PBMCs were added at effector-to-target (E:T) ratios of 5:1 or 3:1 in an equal volume of media. The co-cultures were incubated for 72 hs at 37°C in a humidified incubator with 5% CO₂. After the incubation period, remaining GFP⁺ Panc-1 and mCherry⁺ H460 cells were harvested and quantified using flow cytometry to determine the extent of T cell-mediated killing.

To supplement the flow cytometry assessment, we also quantified the cell abundance via luciferase assay. For cells already in culture, the cell supernatant was removed, and the cells were washed once with PBS. Then, 100 µL of phenol red-free media was added to each well. For frozen cell stocks as positive control, 100 µL aliquots of freshly thawed cells were added to the same plate. Next, 100 µL of firefly luciferase one-step assay solution was added to each well, and the plate was shaken on a plate shaker for 3 mins at medium speed to ensure cell lysis. The plate was incubated for 10 mins at room temperature, after which luminescence was measured using a CLARIOstar plate reader equipped with a luminometer.

### Humanized subcutaneous tumor model in NSG mice

Six-week-old female NSG mice were purchased from the animal core facility at UNC and maintained in a specific pathogen-free facility (12-h light/dark cycle, temperature: 21-23 °C, humidity: 30-70%,). All animal-handling procedures were approved by the Institutional Animal Care and Use Committee at University of North Carolina at Chapel Hill. On day 0, mice were inoculated subcutaneously with either 1 × 10^6^ Panc-1 cells or 1 × 10^6^ H460 in 50ul PBS on their lower flank. Once tumor volume reached ~100 mm^3^ (0.5 × length × width × height), mice were randomized into different groups (n = 5-9) as follows: control IgG +PBMCs, STAB +PBMC, S15 Ab alone+ PBMCs, and CD3 Ab+PBMC and PBMC only. For the treatment, PBMCs were incubated overnight in 50 ml conical tube at 37°C. The cell number was confirmed and injected intravenously in 5E6/200 µl into tumor bearing mice. After 4h, 5µg/mouse STAB antibody in 100 µl PBS was administrated via IV, and the tumor volumes were monitored by caliper every 2 days and calculated as (L×W2)/2, where “L” represents the longer dimension and “W” represents the shorter one. At endpoint of tumor inhibition study, the mice were sacrificed, and tumors were harvested and weighed. Blood was collected on day 0, day 3, day 7 and day 12 for flow cytometry analysis.

### FACS analysis of peripheral blood cells from tumor bearing mice

Blood (50~80 µL) was collected via submandibular bleeding into EDTA tubes, and the blood was mixed immediately to prevent clotting, keeping the tubes at room temperature (RT). The blood was transferred to FACS tubes, and fluorescence-labeled antibodies (listed in Table S1) were added. The samples were incubated at 4°C for 15 mins in the dark. After incubation, 1 mL of BD FACS lysis buffer was added while vortexing, and the samples were incubated for 1 min at RT until the sample became clear. The cells were washed twice with cold FACS buffer (PBS supplemented with 1% FBS and 0.5 mM EDTA). The final pellet was resuspended in 200 µL of FACS buffer for each analysis of a single sample. For intracellular staining using BD Biosciences' protocol, after surface staining, cells are washed twice with cold FACS buffer (PBS with 1% FBS and 0.5 mM EDTA). Cells are then fixed by resuspending in 1-2 mL of BD FACS Lysing Solution for 10 mins at room temperature, followed by two washes with cold FACS buffer. For permeabilization, cells are incubated in 1 mL of BD Perm/Wash Buffer for 15 mins at 4°C in the dark. After permeabilization, intracellular antibodies (e.g., Ki67, TGF-β, IFN-γ) are added and incubated for 30 mins at 4°C in the dark. The cells are washed twice with BD Perm/Wash Buffer, then resuspended in 200 µL of FACS buffer or PBS for flow cytometry analysis.

### Preparation of single cell suspension from tumor tissue

The following instructions are for processing ≤ 1 g of tumor tissue.

Prepare 5 mL of tumor digestion medium by combining the following:

500 μL Collagenase/Hyaluronidase

750 μL DNase I solution (1 mg/mL)

3.75 mL RPMI 1640 medium

Mix thoroughly and warm to room temperature (15 - 25°C).

Mince the tumor tissue into small pieces (≤ 2 mm) using a scissors. The minced tissue was then transferred into a 14 mL round-bottom tube containing tumor digestion medium, and incubated at 37°C for 20 mins on a shaking platform to facilitate enzymatic digestion. Following digestion, the tissue suspension was passed through a 70 μm nylon mesh strainer placed on a 50 mL conical tube. A syringe plunger was used to gently press the digested tissue through the strainer, ensuring optimal single-cell recovery. The strainer was subsequently rinsed with additional RPM1640 medium, and the volume was adjusted to 50 mL. The cell suspension was centrifuged at 300 × g for 10 mins at room temperature. The supernatant was carefully removed and discarded. To lyse residual red blood cells, 1 mL of ACK buffer was added to the pellet and incubated at room temperature for 1 mins. The volume was then adjusted to 50 mL with the RPMI1640 medium, followed by a second centrifugation at 300 × g for 10 mins at room temperature. The supernatant was carefully removed, and the cell pellet was resuspended at a concentration of 1 × 10^6^cells/mL in the RPIM1640 medium.

### Immunofluorescence staining

At the endpoint of the study, mice were sacrificed, and tumors were harvested and prepared for immunohistology staining. After deparaffinizing, antigen retrieval, and permeabilization, tissue sections were blocked in 1% bovine serum albumin (BSA) at room temperature for 1 h. Primary antibodies conjugated with fluorophores (**Table S1**) were incubated overnight at 4°C, and nuclei were counterstained with DAPI-containing mounting medium (Vector Laboratories Inc., Burlingame, CA). All antibodies were diluted according to the manufacturer's manual. Fluorescence images were acquired using Zeiss 880 confocal laser scanning microscope.

### Statistical analyses

Data are expressed as the mean ± SD. GraphPad Prism 10 (GraphPad Software Inc.) was used for statistical analyses. Differences between group means were assessed by Student's t-test (two groups) or (more than two groups) by one-way ANOVA followed with Bonferroni's multiple comparisons test. ***P < 0.001, **P < 0.01, and *P < 0.05 was considered significant.

## References

[1] Becker AE, Hernandez YG, Frucht H, Lucas AL (2014). Pancreatic ductal adenocarcinoma: risk factors, screening, and early detection. World J Gastroenterol.

[2] Di Federico A, Mosca M, Pagani R, Carloni R, Frega G, De Giglio A (2022). Immunotherapy in Pancreatic Cancer: Why Do We Keep Failing? A Focus on Tumor Immune Microenvironment, Predictive Biomarkers and Treatment Outcomes. Cancers.

[3] Bear AS, Vonderheide RH, O'Hara MH (2020). Challenges and Opportunities for Pancreatic Cancer Immunotherapy. Cancer Cell.

[4] Baslan T, Morris JP, Zhao Z, Reyes J, Ho Y-J, Tsanov KM (2022). Ordered and deterministic cancer genome evolution after p53 loss. Nature.

[5] Balachandran VP, Beatty GL, Dougan SK (2019). Broadening the Impact of Immunotherapy to Pancreatic Cancer: Challenges and Opportunities. Gastroenterology.

[6] Adamska A, Domenichini A, Falasca M (2017). Pancreatic Ductal Adenocarcinoma: Current and Evolving Therapies. Int J Mol Sci.

[7] Hosein AN, Dougan SK, Aguirre AJ, Maitra A (2022). Translational advances in pancreatic ductal adenocarcinoma therapy. Nature Cancer.

[8] Hester R, Mazur PK, McAllister F (2021). Immunotherapy in Pancreatic Adenocarcinoma: Beyond “Copy/Paste”. Clinical Cancer Research.

[9] Moreira RS, da Silva MM, de Melo Vasconcelos CF, da Silva TD, Cordeiro GG, Mattos-Jr LAR (2023). Siglec 15 as a biomarker or a druggable molecule for non-small cell lung cancer. Journal of Cancer Research and Clinical Oncology.

[10] Carbone DP, Gandara DR, Antonia SJ, Zielinski C, Paz-Ares L (2015). Non-Small-Cell Lung Cancer: Role of the Immune System and Potential for Immunotherapy. J Thorac Oncol.

[11] Xiao Z, Todd L, Huang L, Noguera-Ortega E, Lu Z, Huang L (2023). Desmoplastic stroma restricts T cell extravasation and mediates immune exclusion and immunosuppression in solid tumors. Nature Communications.

[12] Giraldo NA, Sanchez-Salas R, Peske JD, Vano Y, Becht E, Petitprez F (2019). The clinical role of the TME in solid cancer. British Journal of Cancer.

[13] Johnson A, Townsend M, O'Neill K (2022). Tumor Microenvironment Immunosuppression: A Roadblock to CAR T-Cell Advancement in Solid Tumors. Cells.

[14] Lahiri A, Maji A, Potdar PD, Singh N, Parikh P, Bisht B (2023). Lung cancer immunotherapy: progress, pitfalls, and promises. Molecular Cancer.

[15] Kang FB, Chen W, Wang L, Zhang YZ (2020). The diverse functions of Siglec-15 in bone remodeling and antitumor responses. Pharmacol Res.

[16] Wang J, Sun J, Liu LN, Flies DB, Nie X, Toki M (2019). Siglec-15 as an immune suppressor and potential target for normalization cancer immunotherapy. Nat Med.

[17] Elvin J, Huntington C, Trowsdale J, Barrow A, Cao H (2016). Anti-Siglec-15 antibodies and uses thereof. Google Patents.

[18] Beverley PC, Callard RE (1981). Distinctive functional characteristics of human "T" lymphocytes defined by E rosetting or a monoclonal anti-T cell antibody. Eur J Immunol.

[19] Schepis T, De Lucia SS, Pellegrino A, Del Gaudio A, Maresca R, Coppola G (2023). State-of-the-Art and Upcoming Innovations in Pancreatic Cancer Care: A Step Forward to Precision Medicine. Cancers (Basel).

[20] Principe DR, Underwood PW, Korc M, Trevino JG, Munshi HG, Rana A (2021). The Current Treatment Paradigm for Pancreatic Ductal Adenocarcinoma and Barriers to Therapeutic Efficacy. Front Oncol.

[21] Ligorio M, Sil S, Malagon-Lopez J, Nieman LT, Misale S, Di Pilato M (2019). Stromal Microenvironment Shapes the Intratumoral Architecture of Pancreatic Cancer. Cell.

[22] Wang J, Xu L, Ding Q, Li X, Wang K, Xu S (2023). Siglec15 is a prognostic indicator and a potential tumor-related macrophage regulator that is involved in the suppressive immunomicroenvironment in gliomas. Front Immunol.

[23] Moreira RS, da Silva MM, de Melo Vasconcelos CF, da Silva TD, Cordeiro GG, Mattos-Jr LAR (2023). Siglec 15 as a biomarker or a druggable molecule for non-small cell lung cancer. Journal of Cancer Research and Clinical Oncology.

[24] Zhan W, Bai F, Cai Y, Zhang J, Qin G, Xie Y (2023). Tumor stroma Siglec15 expression is a poor prognosis predictor in colon adenocarcinoma. J Cancer.

[25] Shen L, Schaefer AM, Tiruthani K, Wolf W, Lai SK (2024). Siglec15/TGF-β bispecific antibody mediates synergistic anti-tumor response against 4T1 triple negative breast cancer in mice. Bioengineering & Translational Medicine.

[26] Wang Y, Xu Z, Wu K-L, Yu L, Wang C, Ding H (2024). Siglec-15/sialic acid axis as a central glyco-immune checkpoint in breast cancer bone metastasis. Proceedings of the National Academy of Sciences.

[27] Baeuerle PA, Kufer P, Bargou R (2009). BiTE: Teaching antibodies to engage T-cells for cancer therapy. Curr Opin Mol Ther.

[28] Nagorsen D, Baeuerle PA (2011). Immunomodulatory therapy of cancer with T cell-engaging BiTE antibody blinatumomab. Experimental Cell Research.

[29] Klein C, Brinkmann U, Reichert JM, Kontermann RE (2024). The present and future of bispecific antibodies for cancer therapy. Nature Reviews Drug Discovery.

[30] Zhou S, Liu M, Ren F, Meng X, Yu J (2021). The landscape of bispecific T cell engager in cancer treatment. Biomark Res.

[31] Friedrich MJ, Neri P, Kehl N, Michel J, Steiger S, Kilian M (2023). The pre-existing T cell landscape determines the response to bispecific T cell engagers in multiple myeloma patients. Cancer Cell.

[32] Goebeler M-E, Bargou RC (2020). T cell-engaging therapies — BiTEs and beyond. Nature Reviews Clinical Oncology.

[33] Zhou S, Liu M, Ren F, Meng X, Yu J (2021). The landscape of bispecific T cell engager in cancer treatment. Biomarker Research.

[34] Xu Y, Fu J, Henderson M, Lee F, Jurcak N, Henn A (2023). CLDN18.2 BiTE Engages Effector and Regulatory T Cells for Antitumor Immune Response in Preclinical Models of Pancreatic Cancer. Gastroenterology.

[35] Wathikthinnakon M, Luangwattananun P, Sawasdee N, Chiawpanit C, Lee VS, Nimmanpipug P (2022). Combination gemcitabine and PD-L1xCD3 bispecific T cell engager (BiTE) enhances T lymphocyte cytotoxicity against cholangiocarcinoma cells. Scientific Reports.

[36] Thakur A, Ung J, Tomaszewski EN, Schienschang A, LaBrie TM, Schalk DL (2021). Priming of pancreatic cancer cells with bispecific antibody armed activated T cells sensitizes tumors for enhanced chemoresponsiveness. Oncoimmunology.

[37] Khosla AA, Jatwani K, Singh R, Reddy A, Jaiyesimi I, Desai A (2023). Bispecific Antibodies in Lung Cancer: A State-of-the-Art Review. Pharmaceuticals (Basel).

[38] Shum E, Myint H, Shaik J, Zhou Q, Barbu E, Morawski A (2021). 490 Clinical benefit through Siglec-15 targeting with NC318 antibody in subjects with Siglec-15 positive advanced solid tumors. Journal for ImmunoTherapy of Cancer.

[39] Jin M-Z, Jin W-L (2020). The updated landscape of tumor microenvironment and drug repurposing. Signal Transduction and Targeted Therapy.

[40] Baghban R, Roshangar L, Jahanban-Esfahlan R, Seidi K, Ebrahimi-Kalan A, Jaymand M (2020). Tumor microenvironment complexity and therapeutic implications at a glance. Cell Communication and Signaling.

[41] Neophytou CM, Panagi M, Stylianopoulos T, Papageorgis P (2021). The Role of Tumor Microenvironment in Cancer Metastasis: Molecular Mechanisms and Therapeutic Opportunities. Cancers.

[42] Yang S, Wei W, Zhao Q (2020). B7-H3, a checkpoint molecule, as a target for cancer immunotherapy. Int J Biol Sci.

[43] Getu AA, Tigabu A, Zhou M, Lu J, Fodstad Ø, Tan M (2023). New frontiers in immune checkpoint B7-H3 (CD276) research and drug development. Molecular Cancer.

[44] Heist RS, Christiani D (2009). EGFR-targeted therapies in lung cancer: predictors of response and toxicity. Pharmacogenomics.

[45] Chong CR, Jänne PA (2013). The quest to overcome resistance to EGFR-targeted therapies in cancer. Nature Medicine.

[46] Ayati A, Moghimi S, Salarinejad S, Safavi M, Pouramiri B, Foroumadi A (2020). A review on progression of epidermal growth factor receptor (EGFR) inhibitors as an efficient approach in cancer targeted therapy. Bioorganic Chemistry.

[47] Bose M, Mukherjee P (2020). Potential of Anti-MUC1 Antibodies as a Targeted Therapy for Gastrointestinal Cancers. Vaccines.

