# Supplementary material for: Bispecific Siglec-15/T cell antibody (STAB) activates T cells and suppresses pancreatic ductal adenocarcinoma and non-small cell lung tumors in vivo
Source: Theranostics. 2025 Apr 13;15(12):5529–42. doi: 10.7150/thno.103372 (PMC12068307; doi:10.7150/thno.103372)
Supplement: Supplementary file 1 — Supplementary figures and tables. [file thnov15p5529s1.pdf]

Bispecific Siglec-15/T cell antibody (STAB) activates T cells and suppresses pancreatic ductal adenocarcinoma and non-small cell lung tumors *in vivo*

Limei Shen[1], Alison Schaefer[2], Justin Huckaby[2], Whitney Wolf[1], Samuel K. Lai [1, 3, 4]
[1] Division of Pharmacoengineering and Molecular Pharmaceutics, Eshelman School of Pharmacy, University of North Carolina at Chapel Hill, NC, USA
[2] Department of Biomedical Engineering, University of North Carolina at Chapel Hill, NC, USA
[3] Lineberger Comprehensive Cancer Center, University of North Carolina at Chapel Hill, NC, USA
[4] Department of Immunology and Microbiology, University of North Carolina at Chapel Hill, NC, USA

Supplementary Data

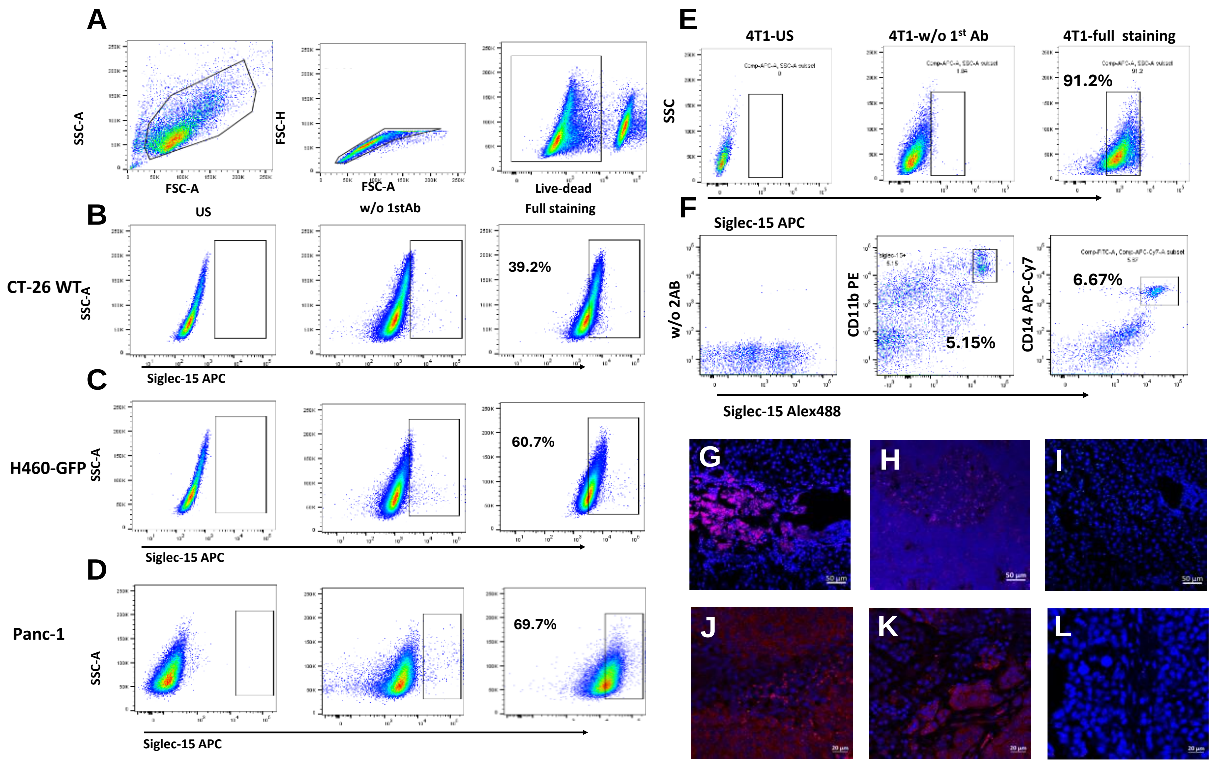

Figure S1. Detection of S15 expression on different cancer cell lines and tumor tissue. (A) Gating strategy. **(B)** CT26 mouse colorectal cancer cell line. **(C)** H460 human NSCLC cell line. **(D)** Panc-1 human pancreatic cancer cell line. **(E)** 4T1 mouse triple negative breast cancer cell line. **(F)** S15 expression on macrophages (CD11b+) and tumor associate fibroblasts (αSMA+). (**G-H**) Primary tumor tissue from 4T1 tumor bearing mouse was stained by anti-S15 antibody-AF647 conjugate in red, (**I**) Negative control (secondary antibody only) for G&H. (**J-K**) Liver with metastasis from 4T1 tumor bearing mice was stained by anti-S15 antibody-AF647 conjugate in red. (**L**) Negative control (secondary antibody only) for J&K.

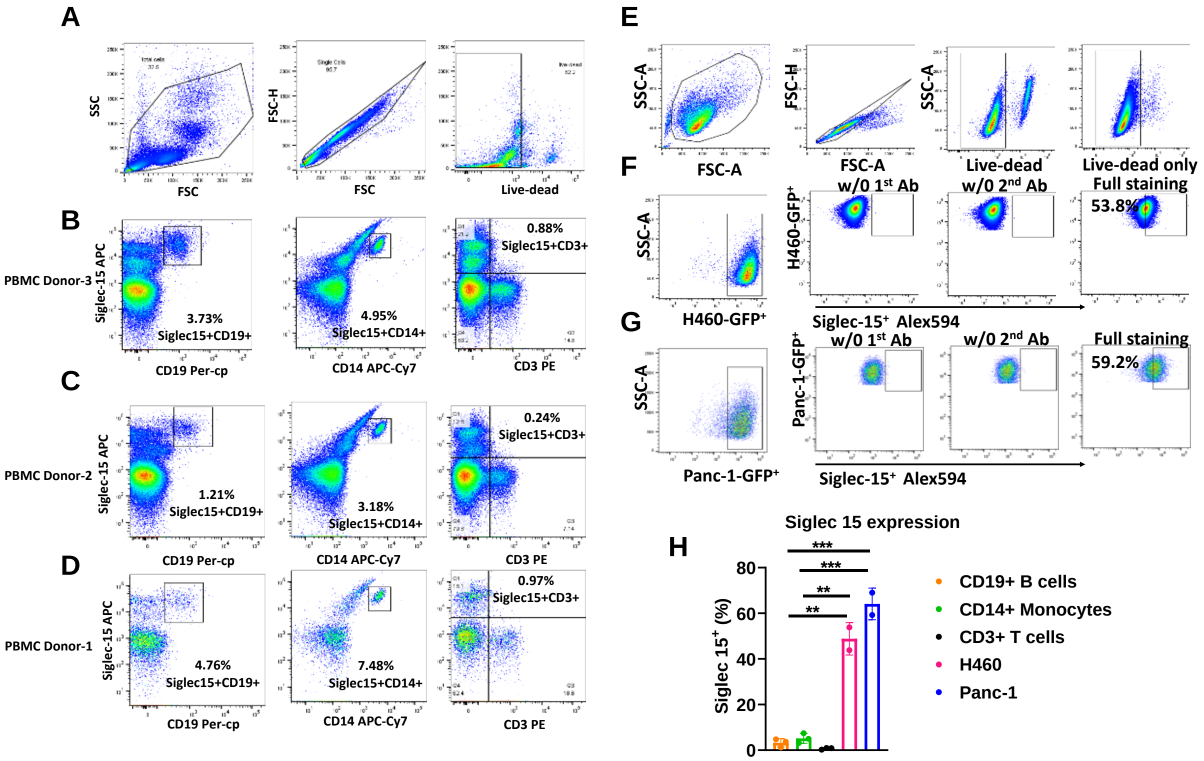

Figure S2. Expression of S15 on various immune cells in PBMC relative to H460 and Panc-1 cancer cell lines. (A) Gating strategy. (**B-D**) Relative abundance of S15+ cells among B cells (CD19+), monocytes (CD14+) and T cells (CD3+) among PBMCs from 3 different healthy donors. (**E**) Gating strategy. (**F&G**) Relative abundance of S15+ (**F**) H460 human NSCLC and (**G**) Panc-1 human pancreatic cancer cell line. (**H**) Relative abundance of S15+ cells between human PBMCs and two cancer cell lines. Statistical differences between groups were performed using two-way ANOVA. Results are presented as mean ± SD. ** $p < 0.01$, *** $p < 0.001$.

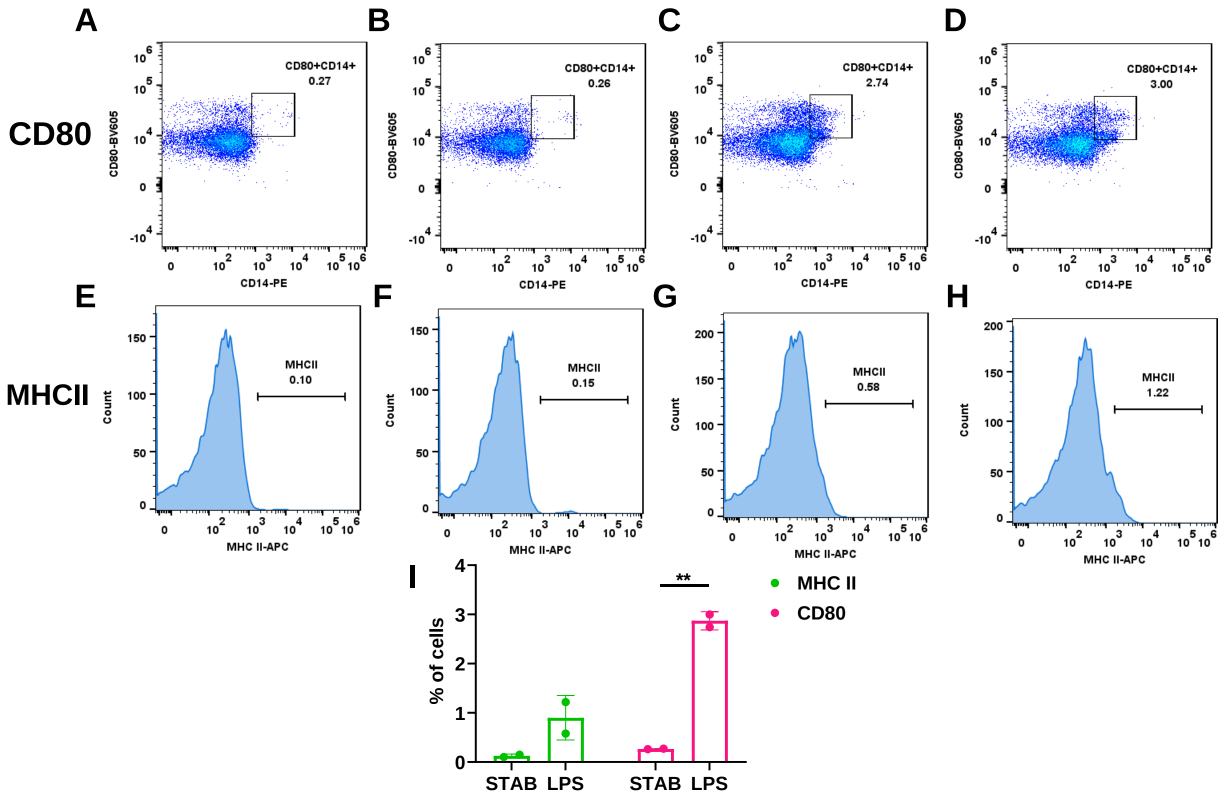

Figure S3. Quantification of MHCII+ (APC) and CD80 (BV605) expression on CD14+ monocytes (PE) analyzed by flow cytometry. PBMCs (0.5 × 10^6 cells) from two donors were incubated with 5 µg/mL STAB or 100 ng/mL LPS at 37°C and 5% CO_2 for 24 hours. PBMCs were then harvested for flow analysis of MHCII and CD80 expression. (**A-D**). Representative flow cytometry plots showing CD80 expression on CD14+ monocytes in PBMCs incubated with (**A, B**) STAB and (**C, D**) LPS. (**E-H**) Representative flow cytometry plots showing MHCII expression on CD14+ monocytes in PBMCs incubated with (**E, F**) STAB and LPS. (**I**) Quantification of flow cytometry results for CD80 and MHCII expression. Statistical differences between STAB treatment and LPS groups were performed using unpair *t*- test. Results are presented as mean ± SD. ***P* < 0.01.

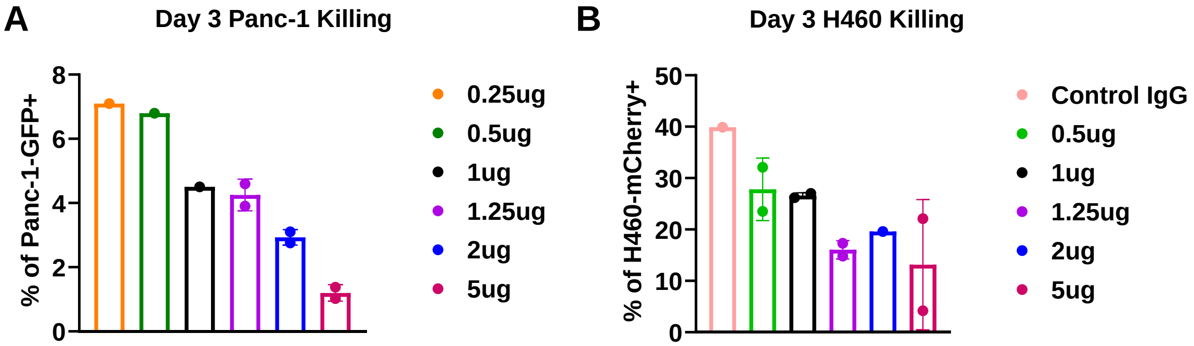

Figure S4. Flow cytometry analysis of tumor killing activity induced by increasing amounts of STAB *in vitro*. PBMCs from two different donors were co-cultured with Panc-1 or H460 tumor cells at a ratio of 3:1 (0.75 × 10^6 PBMCs: 0.25 × 10^6 tumor cells) at different STAB concentrations. After 3 days, the cells were harvested, live-dead staining performed, and GFP+ Panc-1 cells or mCherry+ H460 cells were quantified using flow cytometry. Fraction of total cells that were (**A**) GFP+ Panc-1 or (**B**) mCherry+ H460 cancer cells.

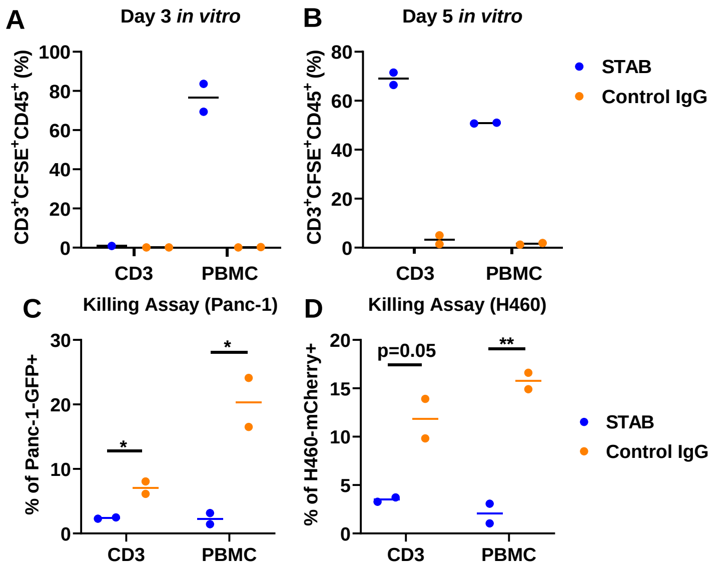

Figure S5. STAB-mediated killing of cancer cells is mediated by CD3+ T cells. CD3+ T cells were isolated from PBMCs of different donors using MACS magnetic column separation. CD3+ T cells and PBMCs were stained with 5 µg CFSE as described in the Methods section. After CFSE staining, STAB or control IgG was added to CD3+ T cells (1.25×10^5 cells) or PBMCs (equivalent number of PBMCs containing CD3+ T cells). After 3 or 5 days, cells were harvested, single-cell suspensions were prepared, and proliferation was analyzed by flow cytometry. CFSE dilution in PBMCs or CD3+ T cells treated with STAB or control IgG on (**A**) Day 3 and (**B**) Day 5. CD3+ T cells and PBMCs from two different donors were co-cultured with Panc-1 or H460 tumor cells at a ratio of 3:1 (0.75×10^5 PBMCs: 0.25×10^5 tumor cells) in the presence of STAB or control IgG. After 5 days, cells were washed and harvested, and flow cytometry were performed. Fraction of (**C**) GFP+ Panc-1 and (**D**) mCherry+ cancer cells from co-cultures with CD3+ T cells or PBMCs. Statistical analyses were performed using two-way ANOVA. Results represent mean ±SD.*$p < 0.05$, ** $p < 0.01$.

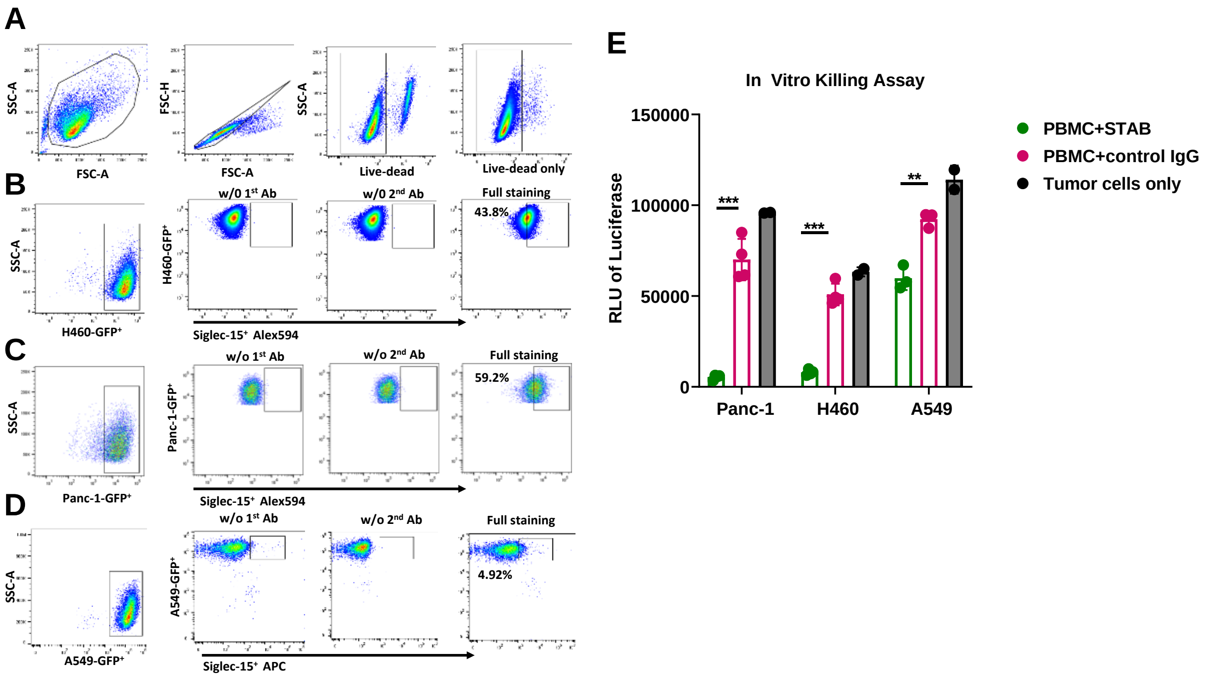

Figure S6. Luciferase assay for assessing STAB-mediated killing of different S15+ cancer cells *in vitro.* (A-D) Evaluation of different expression level of siglec15 on H460+, Panc-1+ and A549+ tumor cells by flow cytometry PBMCs from three different donors were co-cultured with Panc-1, H460 and A549 tumor cells at a ratio of 3:1 (0.75 × 10^6 PBMCs: 0.25 × 10^6 tumor cells). STAB or control IgG were added to the co-culture in 96 well plate and incubated at 37ºC for 72h. Cells were washed 3 days later, resuspended in phenol red-free media, and luminescence measured following addition of luciferin. Results represent mean ± SD. **p < 0.01; ***p < 0.01, statistical analyses were performed using two-way ANOVA by comparison with the control Ig G group.

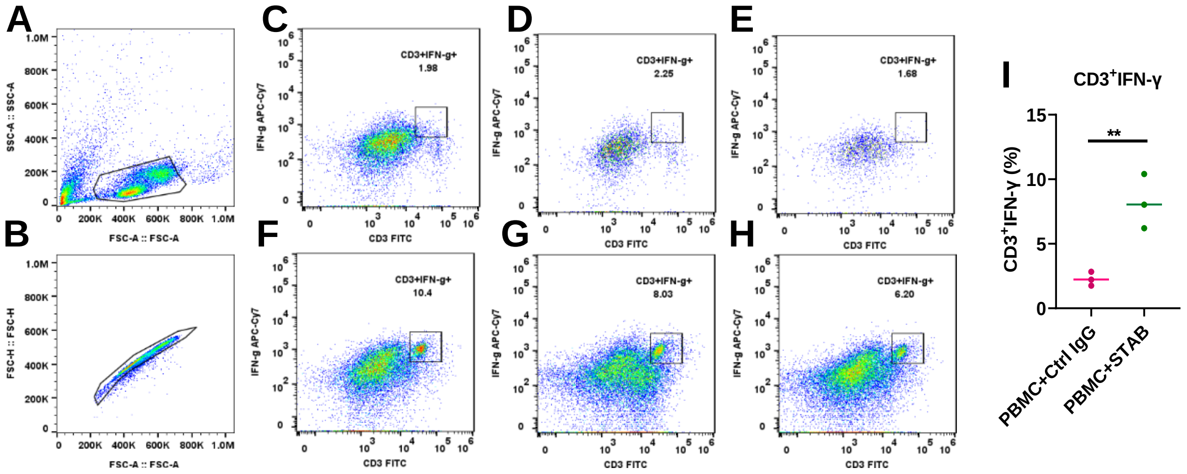

Figure S7: Expression of IFN-γ in tumor-infiltrating CD3+ T cells after STAB treatment. Tumors were harvested at the study endpoint, and single-cell suspensions were prepared as described in the Methods section. Intracellular staining was performed to detect IFN-γ (APC) and CD3 (FITC) in tumor-infiltrating T cells from STAB and control IgG-treated groups. (**A-B**) Gating strategy for identifying IFN-γ$^+$ CD3$^+$ T cells. (**C-E**) Representative flow cytometry plots showing IFN-γ$^+$ CD3$^+$ T cells in the control IgG-treated group (n=3). (**F-H**) Representative flow cytometry plots showing IFN-γ$^+$ CD3$^+$ T cells in the STAB-treated group (n=3).(**I**) Quantification of IFN-γ$^+$ CD3$^+$ T cells from flow cytometry results. Results are presented as mean ± SD. ** p < 0.01. Statistical analyses were performed using nonparametric *t*-test by comparison with the control IgG group.

Reagents	Fluorochrome	Working volume	Catalog #	Clone
BD anti-human CD3	PE	10ul	347347	SK7
BD anti-human CD14	APC-Cy7	2ul	557831	MφP9
Biolegend anti-human CD45	Alex fluro647	2ul	368538	HI30
Invitrogen anti-human Ki-67	FITC	5ul	11-5699-42	SolA15
BD anti-human CD3	PE	10ul/test	555340	SK7
BD anti-human CD19	BV510	5ul/test	562947	HIB19
BD anti-human CD45	APC-Cy7	5ul/test	557833	HI30
BD anti-human CD3	APC-Cy7	1.5ul/test	#557832	SK7
BD anti-human CD19	BV-421	2ul/test	#562440	HIB19
BD anti-human CD14	BV510	2ul/test	#562947	SJ25C1

Table S1. Fluorescently tagged antibody reagents used.

Tumor Tissue sections of human organs, formalin fixed, paraffin embedded (FFPE)					
**Catalog#**	**Organ**	**Age**	**Sex**	**Tissue type**	**Pathology Diagnosis**
HuCAT166	Pancreas	64	F	FFPE	Adenocarcinoma (Grade I)
HuCAT176	Pancreas	65	M	FFPE	Adenocarcinoma (Grade III)
HuCAT171	pancreas	54	F	FFPE	Adenocarcinoma (Grade III)
HuCAT196	Lung Cancer			FFPE	Small cell carcinoma of inferior lobe of right lung
HuCAT206	Lung Cancer			FFPE	Squamous cell carcinoma (Grade I) of superior lobe of right lung
HuCAT226	Lung Cancer			FFPE	Adenocarcinoma (Grade II) of right lung
HuCAT232	Lung Adenocarcinoma	36	M	FFPE	Adenocarcinoma, stage IV

Table S2. Information of all human tumor tissue sections.

Sample	WBC(10^3/µL)	HCT (%)	RBC (10^6/µL)	HGB (g/dL)	PLT (10^5/µL)
Healthy	3.1±0.1	43.8±3.1	10.1±0.7	15.7±1.4	11±0.7
Control AB	5.8±0.7	36.0±0.8	6.5±2.6	10.5±2.8	12.2±3.2
STAB	2.9±0.6	47.7±0.2	9.5±0.9	14.3±0.8	14.4±5.1
Normal range	2.6-10.1	32.8-48	6.5-10.1	10.1-16.1	7.8-15.4

Sample	BUN (mg/dL)	Creatinine (mg/dL)	AST (U/L)	ALT (U/L)
Healthy	12.2±2.2	0.3±0.1	114±31	104±10.4
Control AB	20.8±1.6	0.3±0.2	202.5±40	18.5±6.5
STAB	13.9±2.4	0.4±0.2	146±22	122±11
Normal range	8-33	0.2-0.9	54-298	17-132

Table S3. Toxicological pathology analysis of serum at the endpoint of the study.
